# Extravascular Lung Water and Acute Lung Injury

**DOI:** 10.1155/2012/407035

**Published:** 2011-08-11

**Authors:** Ritesh Maharaj

**Affiliations:** Division of Intensive Care Medicine, King's College Hospital, Denmark Hill, London SE5 9RS, UK

## Abstract

Acute lung injury carries a high burden of morbidity and mortality and is characterised by nonhydrostatic pulmonary oedema. The aim of this paper is to highlight the role of accurate quantification of extravascular lung water in diagnosis, management, and prognosis in “acute lung injury” and “acute respiratory distress syndrome”. Several studies have verified the accuracy of both the single and the double transpulmonary thermal indicator techniques. Both experimental and clinical studies were searched in PUBMED using the term “extravascular lung water” and “acute lung injury”. Extravascular lung water measurement offers information not otherwise available by other methods such as chest radiography, arterial blood gas, and chest auscultation at the bedside. Recent data have highlighted the role of extravascular lung water in response to treatment to guide fluid therapy and ventilator strategies. The quantification of extravascular lung water may predict mortality and multiorgan dysfunction. The limitations of the dilution method are also discussed.

## 1. Introduction

In 1896, the physiologist Starling described the factors that influence fluid transport across semipermeable membranes like capillaries [1]. This description accounted for the net movement of fluids between compartments in relation to capillary and interstitial hydrostatic pressures, capillary and interstitial oncotic pressures, and coefficients of capillary permeability. Pulmonary oedema refers to the accumulation of fluid within the extravascular space of the lung and occurs when the Starling forces are unbalanced. This occurs most commonly from an increased pulmonary capillary hydrostatic pressure or an increased capillary permeability. The estimation of the severity of pulmonary oedema by chest auscultation, radiography, or arterial blood gas analysis is imprecise [2–4]. Chest auscultation may be altered by mechanical ventilation, and bedside chest radiographs in the critical care unit is subject to several technical limitations. There is poor correlation between the chest radiograph scores of pulmonary oedema and the actual amount of EVLW [5]. There is also high interobserver variability when applying the American-European Consensus Conference radiographic criteria for ARDS even amongst experts [6, 7]. Data from experimental studies suggest that EVLW on chest radiography may only be detectable when the lung water increases by more than 35% [8]. Experimental studies have also shown that arterial oxygenation decreased significantly only when the EVLW increases by more than 200% [4]. Hypoxaemia may be due to causes other than pulmonary oedema, and it is estimated that up to one-third of patients with ALI do not have any significant pulmonary oedema [9–11]. The ability to accurately measure EVLW within increments from 10 to 20% offers the potential to identify those patents that may benefit from fluid restriction, dieresis, or other therapies. The aims of this study are to critically analyze clinical studies investigating the prognostic and therapeutic values of EVLW measurement.

## 2. Materials and Methods

Studies were searched in PUBMED by using the terms “extravascular lung water” (EVLW) and “acute lung injury” (ALI) or “acute respiratory distress syndrome” (ARDS) as keywords. The search was further refined by selecting studies investigating the use of dilution methods to assess EVLW in ARDS or ALI. The authors used backward snowballing (i.e., scanning of references of retrieved articles and reviews).

## 3. Results and Discussion

The measurement of EVLW has been under investigation for about 40 years with the first report of the use of EVLW in the clinical management of critically ill patients by Eisenberg and colleagues more than 20 years ago [12]. The bedside method used to measure EVLW was the double-indicator technique. This method requires the simultaneous injection of an intravascular dye indicator and a diffusible (cold saline) indicator. It is assumed that the dye will remain in the intravascular space and the cold temperature will be distributed throughout the thoracic cavity. Differences in the dilution curves allow the calculation of EVLW. The difference in the mean transit time (MTt) multiplied by the cardiac output determine the extravascular thermal distribution volume, that is, intrathoracic thermal volume (ITTV) via cold saline − intrathoracic blood volume (ITBV) via dye dilution = EVLW. This technique is cumbersome, time consuming, and has not been widely used. 

**Figure 1:**
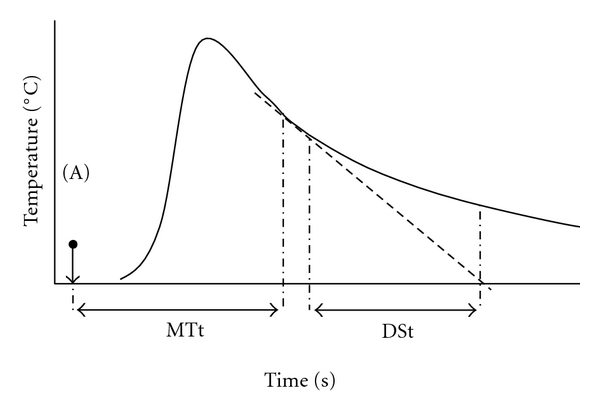
Thermodilution curves showing mean transit time (MTt) and downslope decay time (DSt) of the slope. The time point (A) represents the time of injection. The product of cardiac output and MTt equals the intrathoracic thermal volume. Multiplication of the cardiac output and the DSt equals the pulmonary thermal volume (PTV). The global end diastolic volume (GEDV) is equal to the ITTV − PTV. The intrathoracic blood volume = 1.25 × GEDV. The extravascular lung water (EVLW) is equal to the ITTV less the ITBV.

The single-indicator method uses a thermal indicator to calculate EVLW. Cold saline is injected through a central venous catheter, and the thermistor tip on a femoral arterial catheter, measures the downstream change in temperature in the abdominal aorta. The investigators Sakka et al. were able to quantify the relationship between the ITBV and the global end diastolic volume (GEDV), that is, that total volume and the end of diastole within all four chambers of the heart [13]. The equation is ITBV = 1.25 × GEDV − 28.4 mL. Other investigators have confirmed a similar relationship between ITBV and GEDV [14, 15]. The derivation of EVLW using a single-indicator technique is described in Figure 1 and a more detailed description is available elsewhere [15]. The EVLW represents both interstitial and alveolar fluid. The intrathoracic thermal volume (ITTV) is the product of cardiac output and the MTt. The pulmonary thermal volume is the product of the cardiac output and the exponential downslope time. The difference between the ITTV and the ITBV is an estimate of the EVLW. The accuracy of this technique compares favorably with the gravimetric method, the gold standard test for EVLW [16, 17]. Various formulas relating ITBV to GEDV are reported in Table 1. Importantly, there are no significant differences between the values for ITBV derived from these formulas [18].

## 4. Distinguishing between Cardiogenic and Permeability Pulmonary Oedema

Evidence from several small studies suggests that the ratio of EVLW to ITBV may provide some clue to the cause of pulmonary oedema [19–21]. Early studies used the ratio of the EVLW and the volume of blood within the lungs, that is, the pulmonary blood volume to derive the pulmonary vascular permeability index [22–24]. More recently investigators have attempted to establish the diagnostic value of the EVLW/ITBV ratio [20, 25]. A high EVLW/ITBV ratio would support an increased permeability as the cause, whereas a low ratio would suggest hydrostatic pulmonary oedema. A small study of twenty mechanically ventilated patients by van der Heijden and Groeneveld explored the relationship between pulmonary leak index (PLI) for gallium labeled transferrin and EVLW/ITBV ratios before and after fluid loading in nonseptic patients [26]. The PLI refers to the transvascular transport rate of a protein bound radionuclide, such as ^67^Ga-transferrin or ^99m^technetium-albumin, measured by a bedside probe. A threshold EVLW/ITBV ratio of 0.23 had a positive predictive value of 39% and a negative predictive value of 82% for a high PLI. A similar study in sepsis-related ARDS/ALI found a statistically significant but weak relationship between EVLW and ITBV (*P* = 0.045, *r*_*s*_ = 0.43) [27]. These findings are unsurprising as the sensitivity and specificity of PLI in distinguishing cardiogenic and permeability pulmonary oedema is itself poor. The ITBV may be rapidly reduced when positive pressure ventilation is initiated limiting the interpretation of this ratio. These studies support earlier reports that highlight the potential value and some of the limitations of using EVLW/ITBV ration in trying to distinguish between hydrostatic and permeability pulmonary oedema. The EVLW/ITBV ratio remains an interesting physiologic concept and warrants further clinical enquiry.

## 5. Informing Fluid Therapy

Septic shock and ALI often coexist and require directed interventions. Fluid therapy is an intervention widely applied to almost all critically ill patients and there is consensus that volume resuscitation should occur promptly [28, 29]. Concerns about restoring tissue perfusion must be balanced against the potential harms of volume excess. Simply, being volume responsive does not imply an improved outcome to the administration of volume, and there is some concern about the liberal use of fluids in critically ill patients [30]. A large randomized trial by the ARDS Clinical Trials Network compared liberal and conservative fluid strategies in patients with ALI/ARDS [31]. The 72-hour cumulative for the conservative group was 400 mL and 5100 mL in the liberal group. The study found no difference in mortality at 60 days (25.5% in the restrictive group versus 28.4 for the liberal group, *P* = 0.3). Patients in the conservative group showed an increase in ventilator-free days, reduced ICU stay, no increase in nonpulmonary organ failure, and a trend towards a reduced need for renal replacement therapy. A positive fluid balance in patients with ALI is associated with higher mortality in patients with ALI/ARDS [32]. While these studies did not measure EVLW, it may provide a rational way to monitor patients with ALI/ARDS. Indeed, more than 20 years ago, Eisenberg et al. compared protocol- (EVLW-) guided therapy to routine (pulmonary artery wedge pressure-guided) haemodynamic management in 48 critically ill patients [12]. The study reported a shorter time on mechanical ventilation as well as a lower mortality. The study also showed that restrictive fluid strategies could be safe and well tolerated in patients with ARDS. A follow-up study by Mitchell and colleagues enrolled 101 patients that to the EVLW- and wedge pressure-guided strategies [33]. The EVLW group had a lower positive fluid balance as well as less ventilator days and a shorter ICU stay. 

EVLW has also been used to guide fluid therapy in a cohort of patients with subarachnoid haemorrhage and has been shown to be safe and reduced pulmonary complications [34]. 

ARDS is frequently associated with right heart dysfunction. It is estimated that up to 25% of patients with ARDS may develop acute cor pulmonale (ACP), the most severe form of right ventricular dysfunction [35]. In these patients, fluid administration may exacerbate right ventricular dilatation, worsening ACP. EVLW-guided therapy may offer a method to safely balance resuscitation against the potential harms of fluid excess.

## 6. Titrating PEEP

The consensus view about mechanical ventilation in patients with ALI is to use the appropriate level of PEEP to recruit collapsed lung while delivering small tidal volumes [36]. This simple intervention may have harmful effects on haemodynamic function and gas exchange, and the optimal PEEP has been the subject of substantial investigation [37]. Many techniques have been suggested to titrate the appropriate level of PEEP. These include increasing PEEP to achieve the maximum oxygenation, trading improvements in oxygen delivery against improvements in oxygenation, using pressure volume curves or dynamic stress indices or imaging techniques such as chest radiography and computed tomography [38]. The lack of effect of any of these techniques in large cohorts of patients may reflect the need for a more patient-centered approach. The application of PEEP may affect the measurement of EVLW by dilution methods as well as the actual amount of EVLW [39]. Increasing PEEP may reduce pulmonary vascular flow reducing the measured EVLW [39]. Increasing PEEP may also increase pulmonary flow to previously excluded areas, increasing the measured EVLW [40, 41]. Increasing PEEP may increase the actual EVLW by increasing central venous pressure, creating backward pressure on lymph flow [42]. A decreased pulmonary interstitial pressure may have a similar effect. Increasing PEEP may decrease actual EVLW by decreasing cardiac output, decreasing pulmonary capillary pressure. The application of PEEP may improve oxygenation without significantly changing EVLW. The mechanism in this situation is likely to be recruitment of atelectatic lung. The relationship between PEEP and EVLW remains unsettled and titrating PEEP to individual patients requires consideration of several variables. Comparing pre- and postintervention oxygenation, EVLW and EVLW/ITBV ratios may offer insight into whether the patient has recruited atelectatic lung, haemodynamic changes, pulmonary capillary permeability, and/or hydrostatic forces. This may help to individualize PEEP titration at the bedside.

## 7. Predicting Outcome

Prognostication is an important part of communicating with surrogates and in making decisions about treatment. Despite a wealth of knowledge about ALI and ARDS, prognostication remains difficult [43]. There is considerable overlap between the predictors of mortality in patients with ALI and the predictors of death in the general ICU patient [43]. Age, haematocrit, bilirubin, and 24-hour fluid balance have all been shown to useful clinical predictors from the ARDSNet study [43, 44]. More recently increased EVLW has been identified as a strong predictor or mortality in ALI. Sakka et al. performed retrospective analysis of 373 patients and showed that EVLW was higher in nonsurvivors compared with survivors (median: 14.3 mL/kg versus 10.2 mL/kg, resp., *P* < 0.0001) and predicted mortality independent of SAPS II or APACHE II score by regression modeling [45]. The study also identified a dose effect with mortality lowest in the group with EVLW < 7 mL/kg (<30%), intermediate in the groups <7–14 mL/kg (40%) and 15–20 mL/kg (60%), and highest in the group with EVLW > 20 mL/kg (80% mortality). A threshold of 15 mL/kg was able to discriminate survivors from nonsurvivors (*P* = 0.002). A small prospective study by Kuzkov and colleagues showed a significant correlation between increased EVLW and recognized markers of severity in ALI such as ling compliance, oxygenation ratio, and lung injury score [46]. A significant proportion of patients with ARDS are overweight and EVLW indexed to predicted body weight (PBW) compared with actual body weight (ABW) is higher (20.6 ± 4.6 versus 11.6 ± 1.9 mL/kg; *P* = 0.002) [47]. A 3-day mean EVLW > 16 mL/kg indexed to PBW was found to predict death with 100% sensitivity and 86% specificity. A recent observational cohort study by Craig et al. showed that an elevated EVLW indexed to PBW measured within 48 hours of admission to ICU was significantly associated with mortality [48]. The median EVLW was 17.5 mL/kg (IQR 15.3–21.4) for non-ICU survivors and 10.6 mL/kg (IQR 9.5–15.4) for ICU survivors; *P *<  0.0029. The a odds ratio for death of EVLW indexed to PBW was 4.3 (confidence interval 1.5–2.9) per standard deviation increase, independent of oxygenation index, and APACHE II or SAPS II score. The argument has been that lung volumes are more closely correlated with height and gender than with actual body weight. Therefore, indexing EVLW to obese patients is likely to underestimate the severity of pulmonary oedema. An elevated EVLW indexed to PBW also predicts the development of multiorgan dysfunction syndrome (MODS) [49]. Data from small studies support the role of EVLW in predicting the clinical behavior during mechanical ventilation. High-frequency ventilation is better tolerated in patients with an EVLW > 15 mL/kg and pressure support better tolerated when the EVLW < 11 mL/kg [50, 51]. While the signal from these small, single center studies is encouraging, there is a lack of consensus about the definition of the normal values for EVLW or whether EWLV should be indexed to PBW or ABW.

## 8. Limitations of EVLW

It is estimated that up to one-third of patients with ALI/ARDS criteria do not have significant pulmonary oedema [9, 52, 53]. The mechanism for hypoxaemia in this group of patients may be due to atelectasis or consolidation in these patients. The diagnosis of ALI/ARDS on clinical criteria does not correlate well with autopsy findings [54]. EVLW may offer a reliable means of characterising ALI/ARDS by identifying those patients with increased pulmonary vascular permeability. This offers the prospect of a more homogenous group of patients that may benefit from interventions such as fluid restriction and diuresis and to recruit for further clinical trials. The measurement of EVLW may be altered by systematic or accidental errors of measurement. The single-indicator method relies on a predictable and constant relationship between the GEDV and the ITBV. Underperfusion that occurs pulmonary resection, pulmonary embolism, and pulmonary arterial occlusion may underestimate EVLW by about 10% [55, 56]. Experimental evidence suggests that this observation occurs only when vessels with a diameter >500 *μ*m are occluded [57]. High cardiac output states may not allow sufficient time for equilibration with the extravascular distribution volume. Michard et al. studied the effect of cardiac index changes in critically ill patients in the range 1.9 L/min/m^2^ to 7.1 L/min/m^2^ as well as hyper- an hypovolaemic states but found no change in the relationship between GEDV and ITBV [15]. The presence of an intra-aortic balloon pump (IABP) renders continuous pulse contour analysis inaccurate but does not affect the accurate estimation of EVLW [58]. The same applies for the concomitant use of renal replacement therapy. EVLW calculation is accurate, provided that the vascular access catheter is not in the path of the indicator. A large aortic aneurysm may lead to underestimation of the EVLW and intracardiac shunts have unpredictable effects on the measured EVLW [59].

## 9. Conclusion

The use of thermodilution techniques to assess EVLW provides an accurate and readily accessible method at the bedside in critically ill patients. EVLW may have value in decision making about titrating PEEP, predicting clinical behavior during mechanical ventilation, guiding fluid therapy, and manipulating fluid balance and ultimately about prognostication.

## Figures and Tables

**Table 1:** Different formulas for the calculations of ITBV reported in the current literature.

PiCCO monitor	ITBV = 1.25 × GEDV
Sakka et al. [13]	ITBV = 1.25 × GEDV − 28
Reuter et al. [14]	ITBV = 1.16 × GEDV + 97
Michard et al. [15]	ITBV = 1.10 × GEDV + 180
